# Linezolid administration to lactating Wistar rats affects the health of their offspring

**DOI:** 10.1007/s00210-025-04060-8

**Published:** 2025-04-12

**Authors:** Aya G. Hamouda, Entsar R. Abd-Allah, Aya A. Mahmoud

**Affiliations:** 1 Zoology Department, Faculty of Science, Cairo University, Giza, Egypt; 2 Zoology Department, Faculty of Science, Al-Azhar University, Nasr City, Egypt

**Keywords:** Linezolid, Wistar rat, Lactation, Oxidative stress, Body weight, Comet assay, Offspring

## Abstract

Lactational exposure to antibacterial medications may affect the normal development of newborns during this crucial stage and later in adult life. Linezolid (LNZ) is an oxazolidinone antibacterial drug that is effective against drug-resistant Gram-positive bacteria and multidrug-resistant *Mycobacterium tuberculosis*. Although it is relatively toxic, there is insufficient data about LNZ use during lactation. This study aimed to elucidate the impact of linezolid administration during lactation on Wistar rats’ offspring. Eighteen lactating Wistar female rats were separated into three groups (*n* = 6): control, therapeutic, and low dose groups. The therapeutic dose group received 61.66 mg/kg of LNZ (equivalent to the human dose), while the low dose group received 15.41 mg/kg of LNZ (1/4 of the human therapeutic dose) by gavage twice daily. All lactating dams and their offspring died four days after receiving a therapeutic dose. In the low dose group, LNZ significantly reduced the body weight of lactating females and their pups. The liver tissue of the pups showed a considerable increase in malondialdehyde levels, along with a decrease in the catalase, glutathione, and superoxide dismutase activities accompanied by moderate histological alterations like congestion, and infiltration, and DNA fragmentation as indicated by comet assay. Microscopic examination of renal tissue revealed glomeruli deterioration, cellular infiltration, and intratubular protein deposits. In conclusion, this study highlights the potential risks linezolid may pose to infants during postpartum. Therefore, there is a need for preweaning monitoring and caution should be taken during breastfeeding.

## Introduction

Maternal health during breastfeeding period is a crucial factor for proper development and growth of neonates (Kominiarek and Rajan 2016). Antibacterial drugs are frequently prescribed for mothers during lactation. It was previously estimated that approximately 40% of pregnant women receive an antibacterial drug during pregnancy and immediately postpartum (Nahum et al. 2006; Ledger and Blaser 2013;Shao et al. 2019) to prevent postpartum infection arising from trauma received during birth, post-surgical problems after a cesarean section, or physiological changes that may develop throughout pregnancy (Stokholm et al. 2013; Persaud et al. 2015).

Antibacterial drugs prescribed for breastfeeding women are presumed to be safe for newborns, allowing mothers to continue breastfeeding during these treatment periods (Kaiser et al. 2007). However, antibacterial drugs administered to the dam may pass through breast milk and potentially disturb the delicate balance of the newborn microbiota, increasing the risk of adverse health outcomes (Tamburini et al. 2016). Studies on mice have shown that maternal antibacterial treatment during the perinatal period has long-term impacts on the pups’ metabolic activity (Cox et al. 2014) and immunity (Russell et al. 2013).

Oxazolidinones are artificial antibacterial drugs that have reached clinical trials in the past fifty years (Foti et al. 2021). Oxazolidinones have gained popularity because of their exceptional mechanism of action, which ensures strong antibacterial potency while reducing susceptibility to mechanical resistance against several types of multidrug-resistant Gram-positive bacteria (Foti et al. 2021). They inhibit protein production by binding at the P site of the ribosomal 50S subunit. Resistance to other protein synthesis inhibitors does not affect oxazolidinone activity. However, rare development of oxazolidinone resistance cases associated with 23S rRNA alterations during treatment have been reported (Bozdogan and Appelbaum 2004).

Linezolid (LNZ) is an oxazolidinone bacteriostatic antibacterial drug that targets drug-resistant Gram-positive bacteria such as vancomycin-resistant *Enterococcus*, methicillin-resistant *Staphylococcus aureus*, and multidrug-resistant *tuberculosis* (Hashemian et al. 2018; Crass et al. 2019). It inhibits bacterial growth and reproduction by interrupting mRNA translation into proteins in bacterial ribosomes at the initiation stage, unlike most other protein synthesis inhibitors, which impede elongation. The mechanism of action relies on blocking the development of the initiation complex by binding to the 23S region of the 50S subunit, which is the site of peptidyl transferase activity (Zhanel et al. 2015; Roger et al. 2018). The Food and Drug Administration has approved LNZ for a variety of diseases, including the treatment of complex skin and skin structure infections caused by *Streptococcus pyogenes* or methicillin-sensitive *Streptococcus aureus*, as well as community-acquired pneumococcal meningitis caused by penicillin-resistant *Streptococcus pneumoniae* (Hughes et al. 2015; Tang et al. 2015). Its absolute bioavailability allowed the change from intravenous to oral administration without dose modification (Roger et al. 2018). Rapid absorption to reach peak plasma concentrations 1 to 2 h following drug administration was recorded in healthy adult volunteers (Stalker and Jungbluth 2003). These attributes ensure the efficacy of drug levels in the body, rendering LNZ a promising therapeutic option for treating serious bacterial infections (Zou et al. 2024).

The most common adverse reactions linked to LNZ usage are headache and gastrointestinal disorders such as nausea, diarrhea, and vomiting (Esmail et al. 2022; Kotsaki et al. 2022). Studies have shown that it has been related to serious side effects, including peripheral and optic neuropathy at a dose of 600 mg per day for six months (Karuppannasamy et al. 2014), lactic acidosis (Carbajo et al. 2011), and bone marrow suppression (Bishop et al. 2006) which include disorders such as anemia and thrombocytopenia (Dai et al. 2021). Furthermore, LNZ suppresses cell proliferation and slow down cellular metabolic activity by affecting mitochondrial function (Duewelhenke et al. 2007).

Anti-ribosomal antibacterial drugs are associated with drug-induced mitochondrial dysfunction due to structural similarities between bacterial and mitochondrial ribosomes. Mitochondrial dysfunction has been identified as a significant cause of drug-induced liver damage (Kiy 2024). Vinh and Rubinstein (2009), Vivekanandan et al. (2018), and Abd El Latif et al. (2019) observed that LNZ intake is linked to alterations in the levels of liver enzymes.

Linezolid has been shown to alter antioxidant/oxidant status by increasing lipid peroxidation and decreasing the activity of antioxidant enzymes in blood and liver of LNZ-treated rats, resulting in liver damage (Wang et al. 2014; Vivekanandan et al. 2018; Abd El Latif et al. 2019). Furthermore, Wang et al. (2016) found that LNZ-treated patients had higher oxidative stress indicators, which could be the underlying mechanism of induced thrombocytopenia.

LNZ clearance varies with age and gender; it is fastest in children (resulting in a shorter half-life) and appears to be 20% lower in women than in men (Slatter et al. 2001; Sisson et al. 2002). Under steady-state conditions, approximately 30% of the administered dose is eliminated in the urine as linezolid, while 50% is excreted in the form of linezolid metabolites, which can accumulate in patients with renal impairment (Dryden 2011). Elevation in urea and creatinine serum levels was previously recorded after LNZ treatment (in the recommended dosage and duration), hence causing renal impairment (Takahashi et al. 2011; Abdel Aziz et al. 2017; Vivekanandan et al. 2018; Hindawy and Hendawy 2019).

Linezolid is classified as a pregnancy category C medication. Rats and mice exposed to LNZ at four times the estimated human dose did not exhibit teratogenic effects (Hillard 2002; Navarro et al. 2023). However, because of its minimal protein binding (~ 30%), small molecular weight (337 Daltons), and elevated oral bioavailability (100%), LNZ has the potential to reach the systemic circulation of suckling infants in clinically meaningful quantities (Pfizer 2014). In accordance with the product profile, LNZ and its metabolites are released in rat milk at concentrations comparable to maternal plasma (Pfizer 2014). Adverse toxic effects, including post-implantation embryonic mortality, decreased fetal weight, and decreased survival rate during the early postnatal days, were detected in pups of female rats treated with LNZ during gestation and lactation periods (Hillard 2002).

Recently, Li et al. (2024) noted that older patients receiving a standard dose of linezolid (600 mg) were associated with increased health risk and recommended a modified dosage of 300 mg every 12 h. Furthermore, a study conducted by Abouelkheir et al. (2024) highlighted the complex interplay of risk factors for linezolid-associated hematologic toxicity. It emphasized the importance of careful monitoring, especially in pediatric patients.

Numerous studies have demonstrated the adverse effects of linezolid administration in rats and humans (Hillard 2002; Abdel Aziz et al. 2017; Hindawy and Hendawy 2019; Abd El Latif et al. 2019; Kendir-Demirkol et al. 2023; Abouelkheir et al. 2024; Li et al. 2024). However, the literature currently lacks evidence on the toxic impacts of LNZ on pups’ health during lactation. Since the long-term consequences of antibacterial drugs on infants’ health depend on timing, this experimental design seeks to investigate the potential adverse effects of maternal LNZ administration during lactation on Wistar rat’s offspring by assessing the oxidative stress biomarkers, hepatic and renal histopathology, and DNA damage and to evaluate the safety of its usage by nursing mothers during this vital period.

## Material and methods

### Linezolid (LNZ)

LNZ (100 mg/5 ml powder for oral suspension) was purchased from Averroes Pharma Company, Egypt, under the trade name Averozolid®. The powder was reconstituted in 5 ml distilled water to obtain a concentration of 20 mg/1 ml uniform suspension. Doses were prepared shortly before treatment and adjusted weekly according to the change in body weight of lactating rats.

### Experimental animals

Nine males (weighing 170–190 g) and eighteen female Wistar rats (weighing 160–180 g) were purchased from Egypt’s National Research Center. Two females were mated with a male and eighteen pregnant rats were separated and housed individually during their gestation. After parturition, 18 lactating females and their offspring were divided into three groups, each with six pups assigned to a lactating dam and housed separately for 21 days (lactation period). Rats were housed in a regulated 12-h dark/light cycle at a temperature of 22 ± 1 °C and relative humidity of 55 ± 5%. All animals were allowed to adapt to a laboratory environment with free access to food and water for one week prior to the onset of the experiment (Basal et al. 2020). All experiments and procedures followed the relevant international guidelines for the care and use of laboratory animals. The Institutional Animal Care and Use Committee (IACUC), Faculty of Science, Cairo University, approved the experiments (approval number; CU/I//F/13/23).

### Dose calculations

LNZ was orally administered at two concentrations: therapeutic dose (equivalent to human therapeutic dose (600 mg/adult person/12 h) (Pfizer 2014) and low dose (one-fourth of the human therapeutic dose).

Animal dose was calculated as follows (Nair and Jacob 2016):

$$\text{Animal dose}=\text{Human dose }\times \frac{\text{Human correction factor }(\text{KmH})}{\text{Animal correction factor }(\text{KmA})}$$

$$\text{Animal dose}=\frac{600}{60}\times \frac{37}{6}$$

Thus, the calculated doses were 61.66 mg/kg/12 h for the therapeutic dose and 15.41 mg/kg/12 h for the low dose.

### Experimental design

The current study is designed to evaluate the impact of maternal treatment with the therapeutic dose of LNZ during breastfeeding on Wistar rat offspring and to assess the safety of using a lower, subtherapeutic dose. Eighteen lactating rats (6 rats/group) and their offspring (6 pups/dam) were separated into three groups, which received oral treatments twice daily at the same time during the lactation period (from postnatal day 1 to 21) as follows:

- Control group: Lactating dams received distilled water.
- Therapeutic dose group: Lactating dams were administered 61.66 mg/kg/12 h of LNZ (French 2003; Pfizer 2014).
- Low dose group: Lactating rats were administered 15.41 mg/kg/12 h of LNZ.

The body weight of mothers and their pups was recorded weekly. At the end of the trial, pups from all lactating dams were anesthetized with 50 mg/kg of sodium pentobarbital intraperitoneally and sacrificed (Mahmoud et al. 2024). The offspring’s liver and kidney tissues were collected for histopathological examination. Evaluation of oxidative stress biomarkers (MDA, SOD, GSH, and CAT) and DNA damage using comet assay were assessed in pups’ liver tissue.

### Histopathological examination

Liver and kidney tissue were removed from pups of all experimental groups and immediately fixed in 10% neutral buffered formalin for 24 h. Preparation of paraffin sections and hematoxylin–eosin staining were performed according to the method previously described (Bancroft et al. 2013). All sections were examined using a BX53M light microscope (Olympus, Japan) and photographed. The score of histopathological changes in liver and kidney tissues were recorded as follows: ( −) normal, ( +) mild, (+ +) moderate, and (+ + +) severe (Derelanko 2008).

### Estimation of oxidative stress markers

Frozen liver tissues were homogenized with cold phosphate buffer (1:10 ml) and centrifuged at 12,000 rpm for 20 min. The supernatant was separated to assess oxidative biomarkers. Biodiagnostic kits (catalogue numbers; MD2529, CA2517, GR2511, and SD2521) were purchased to evaluate MDA, CAT, GSH, and SOD levels, respectively, following manufacturer instructions. UV-2100 spectrophotometer (Quality Test, USA) was used to measure liver MDA, CAT, GSH, and SOD levels, following the procedures described by Ohkawa et al. (1979), Aebi (1984), Beutler et al. (1963), and Nishikimi et al. (1972), respectively.

### Assessment of DNA fragmentation by comet assay

The alkaline comet assay, previously reported by Tice et al. (2000) was used to determine the degree of DNA strand breaks in the liver tissues of both the control and treated groups. The comet analysis was carried out using a Carl Zeiss Axio fluorescence microscope (Germany). The microscope is equipped with a 524 nm excitation filter and a 605 nm barrier filter. The Komet 5.0 analytic system, created by Kinetic Imaging, Ltd. (Liverpool, UK) was utilized with a charge-coupled device (CCD) camera to quantify the proportion of % of damage, DNA % in tail, tail length, and tail moment.

### Statistical analysis

Independent sample *t*-tests were performed using the SPSS software program (version, 19.0; IBM SPSS, Armonk, NY, USA). Data were stated as mean ± standard error of the mean. A *P* < 0.05 was regarded as a statistically distinct change.

## Results

### Effect of therapeutic dose on lactating females and their pups

The results showed that all lactating dams (*n* = 6) who orally administered the therapeutic dose of LNZ (61.66 mg/kg twice daily) and their pups (*n* = 36) were lost after four days of dosing compared to the low dose (15.41 mg/kg LNZ) and control groups. This means that the therapeutic dose is lethal for lactating dams and their pups.

### Effect of linezolid administration on the body weight of lactating rats and their pups

In the low dose group, it was found that all lactating dams (6/group) with their pups (36/group) completed the period of lactation without any loss. By the end of week 1, lactating females treated with a low dose of LNZ demonstrated a statistically (*P* < 0.05) significant decrease in body weight compared to those in the control group. The weight drop continued until the end of the lactation period (Table 1). In pups of low dose LNZ-treated dams, despite gaining weight, their body weights were significantly (*P* < 0.05) lower than their peers in the control group (Table 2).

**Table 1:** Effect of LNZ on maternal body weight (g) during lactation

Day/group	Control	Low dose (15.41 mg/kg)
Number of dams	6	6
1st day	197.16 ± 2.93	197.6 ± 2.13
7th day	219.16 ± 2.59	196.4 ± 2.61^a^
14th day	229.25 ± 1.81	195.6 ± 2.50^a^
21st day	241.75 ± 2.42	178.6 ± 5.20^a^

Each value represented as means ± SEM

^a^Letter means there was a significant difference (*P* < 0.05) as compared with control group

**Table 2:** Effect of LNZ maternal administration on pup’s body weight (g) during lactation

Day/group	Control	Low dose (15.41 mg/kg)
1st day	5.53 ± 0.42	5.87 ± 0.20
7th day	11.15 ± 0.40	8.86 ± 0.84^a^
14th day	23.44 ± 0.70	18.44 ± 0.81^a^
21st day	41.29 ± 2.27	30.11 ± 1.90^a^

Each value represented as means ± SEM. *n* = 36

^a^Letter means there was a significant difference (*P* < 0.05) as compared with control group

### Effect of LNZ on pups’ organs weight

LNZ treatment of lactating rats with 15.41 mg/kg/12 h showed no significant (*P* > 0.05) decrease in the offspring’s liver and kidney weights compared to the control group (Table 3).

**Table 3:** Effect of LNZ on rat pup’s organ weight (g) during lactation

Organ/group	Control	Low dose (15.41 mg/kg)
Liver	2.01 ± 0.19	1.51 ± 0.56
Right kidney	0.30 ± 0.03	0.25 ± 0.06
Left kidney	0.30 ± 0.02	0.20 ± 0.04

Each value represented as means ± SEM. *n* = 36

### Oxidative stress markers

As recorded in Table 4, the levels of malondialdehyde (MDA) were significantly (*P* < 0.05) elevated in liver supernatants obtained from pups of low dose LNZ-treated lactating rats compared to the control, while the antioxidant activities of CAT, GSH, and SOD were significantly (*P* < 0.05) decreased in the low dose LNZ-treated group relative to the control group.

**Table 4:** Oxidative stress markers of pups after LNZ treatment of lactating females

Parameter/group	Control	Low dose (15.41 mg/kg)
MDA (nmol /g tissue)	23.86 ± 0.68	31.27 ± 0.77^a^
CAT (U/g tissue)	2.97 ± 0.08	1.97 ± 0.04^a^
GSH (mg/g tissue)	91.6 ± 0.92	79.3 ± 0.55^a^
SOD (U/g tissue)	119.77 ± 2.87	71.63 ± 0.56^a^

Each value represented as means ± SEM

^a^Letter means there was a significant difference (*P* < 0.05) as compared with control group. *n* = 36

### Histological examination

#### Liver histopathology

**Fig. 1:**
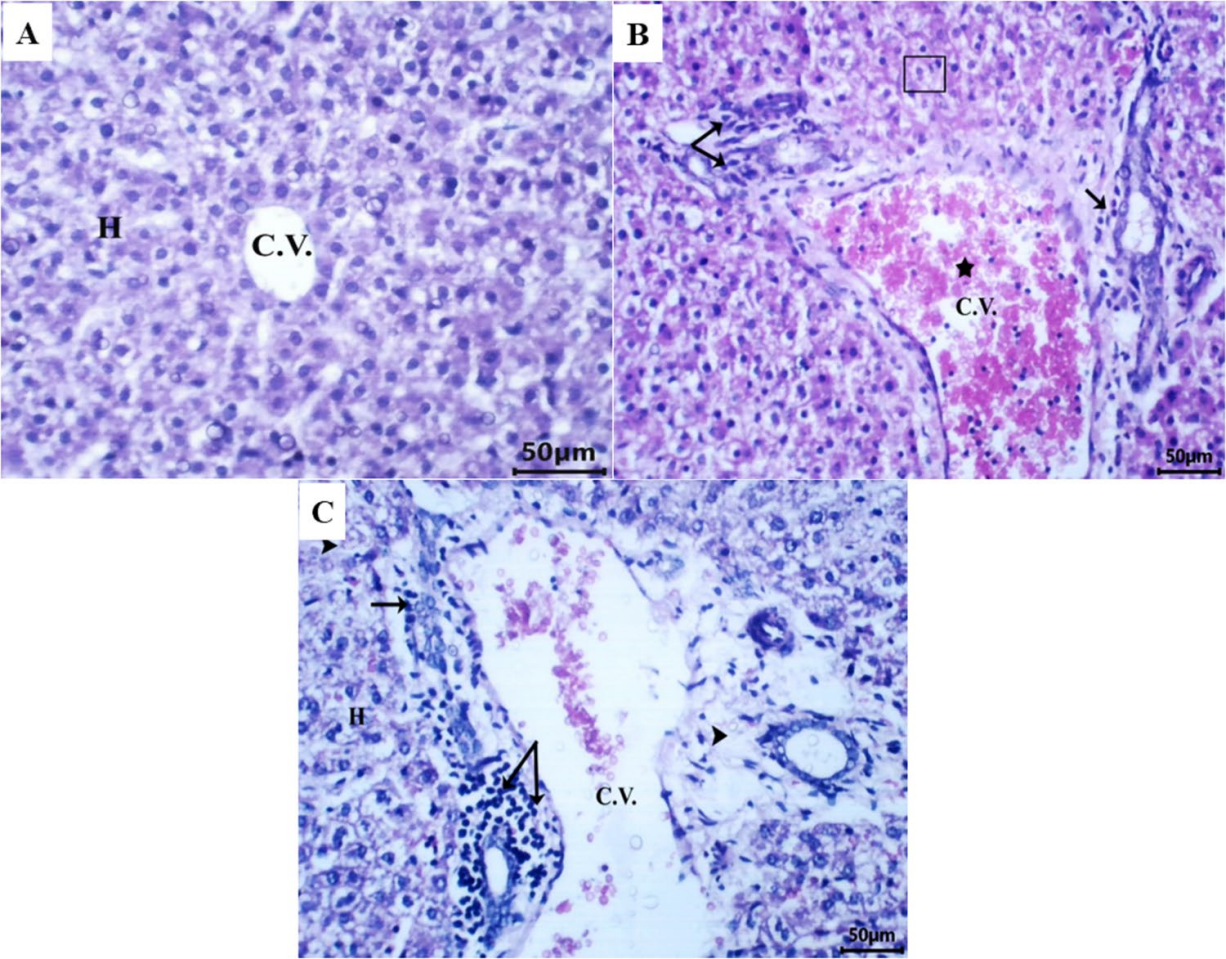
Photomicrograph of pup’s liver tissue sections stained with hematoxylin and eosin obtained from control and low dose groups. **A **Control group: demonstrated normal hepatocytes and their nuclei (H) around a central vein (C.V.). **B **Low dose (15.41 mg/kg LNZ) group: showed loss of hepatocytes architecture (cube), dilated central vein (C.V.) with congestion (star) and infiltration (arrow). **C **Low dose (15.41 mg/kg LNZ) group: showed degenerated hepatocytes region (arrowhead) and infiltration (arrow)

Table 5 and Fig. 1 illustrate histological alterations of hepatic tissue in the control group and the lesion severity in the 15.41 mg/kg LNZ-treated group. Liver tissue sections of the control group’s pups revealed normal hepatocytes and nuclei surrounding a central vein (Fig. 1A). The pup’s liver sections of the low dose group showed loss of hepatocyte architecture, moderate central vein dilation, and mild hepatocyte damage. In addition, moderate congestion and infiltration were observed (Fig. 1B, C).

**Table 5:** The severity of the histopathological alterations in liver tissue of pups of control and low dose groups

Histopathological alteration/group	Control	Low dose (15.41 mg/kg)
Damaged hepatocytes area	-	+
Congestion	-	+ +
Dilated central vein	-	+ +
Infiltration	-	+ +

(+ + +) Severe, (+ +) moderate, ( +) mild, and (–) normal. *n* = 36

### Kidney histopathology

**Fig. 2:**
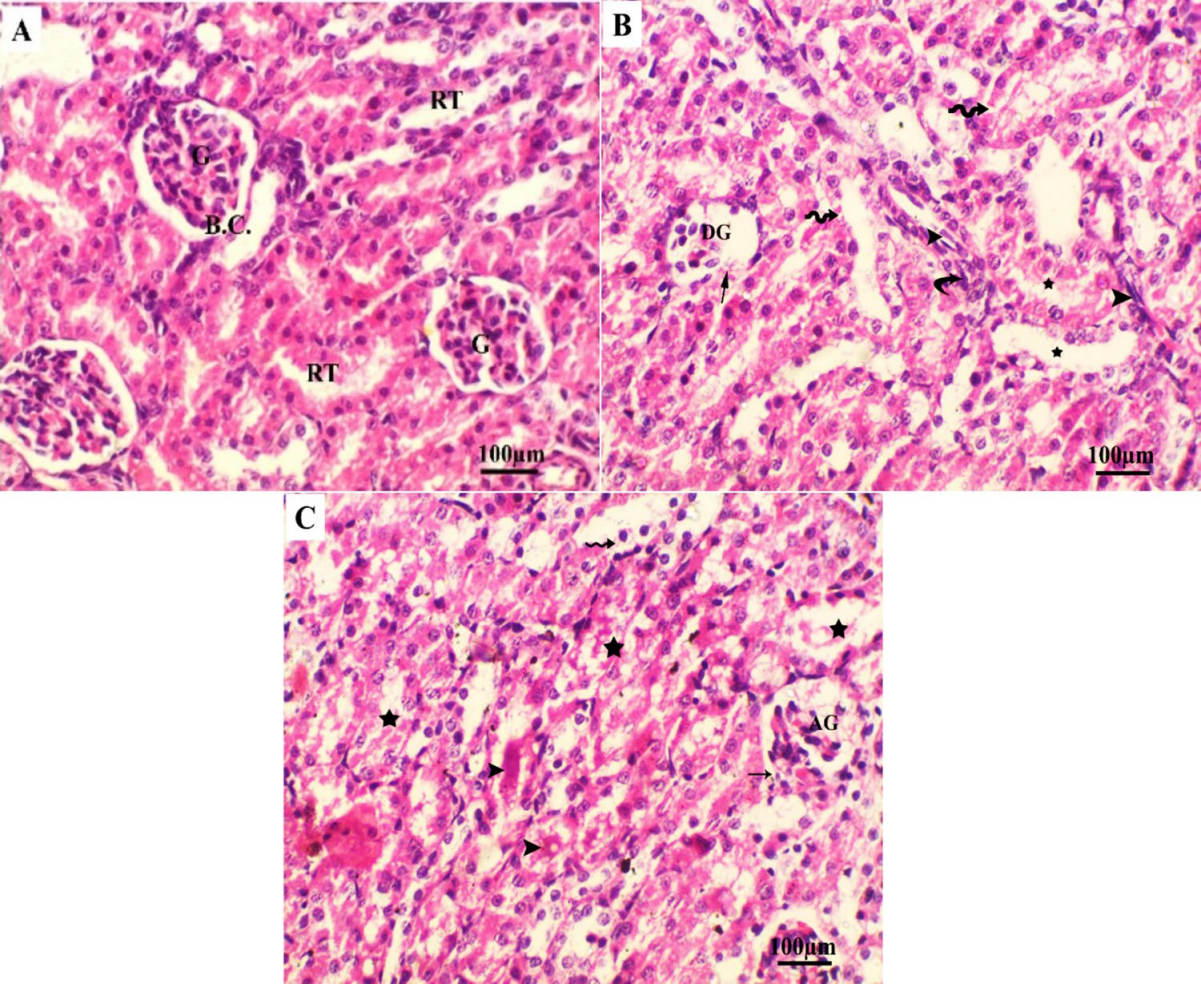
Photomicrograph of pup’s kidney tissue sections stained with hematoxylin and eosin obtained from control and low dose groups. **A **Control group: showed a regular histological pattern of renal tissues. Normal glomeruli (G) surrounded by Bowman’s capsule (B.C.) in addition to normal renal tubules (RT). **B **Low dose (15.41 mg/kg LNZ) group: showed completely degenerated glomerulus (DG) surrounded by partial deterioration of Bowman’s capsule basement membrane (arrow), fragmented tubular basement membrane (wavy arrow), infiltrated with metaplastic epithelial cells (arrowhead), hyperplasia (curved arrow) and damaged renal tubules (star). **C **Low dose (15.41 mg/kg LNZ) group: demonstrated Bowman’s capsule partially fragmented (arrow) encapsulated atrophied glomerulus (AG), intratubular accumulation of protein (hyaline) casts (arrowhead), loss of tubular epithelial lining (wavy arrow), damaged (star) renal tubules

Table 6 and Fig. 2 show the histological changes in renal tissue and the severity of lesions in the low dose LNZ-treated group. Kidney tissue sections from control pups revealed a consistent histological pattern of renal tissues. Bowman’s capsule surrounds normal glomeruli as well as normal renal tubules (Fig. 2A). Kidney tissue sections from pups of the low dose-treated group revealed severe changes, including partially fragmented Bowman’s capsule encapsulating deteriorated and atrophied glomeruli (Fig. 2B, C). Furthermore, renal tubules showed moderate loss or fragmentation of the tubular basement membrane, infiltration with metaplastic epithelial cells, hyperplasia, and renal tubule damage (Fig. 2B, C). Moderate intratubular deposition of protein (hyaline) casts was also observed (Fig. 2C).

**Table 6:** The severity of the histopathological alterations in kidney tissue of control and low dose groups

Histopathological alteration/group	Control	Low dose (15.41 mg/kg)
Degenerated glomeruli	-	+ + +
Atrophied glomeruli	-	+ + +
Deteriorated Bowman’s capsule	-	+ + +
Protein casts	-	+ +
Loss or fragmented tubular basement membrane	-	+ +
Infiltrated tubule with metaplasia	-	+ +
Tubular hyperplasia	-	+ +
Damaged renal tubules	-	+ +

(+ + +) Severe, (+ +) moderate, ( +) mild, and (–) normal. *n* = 36

### Assessment of the genotoxicity of LNZ by comet assay

**Fig. 3:**
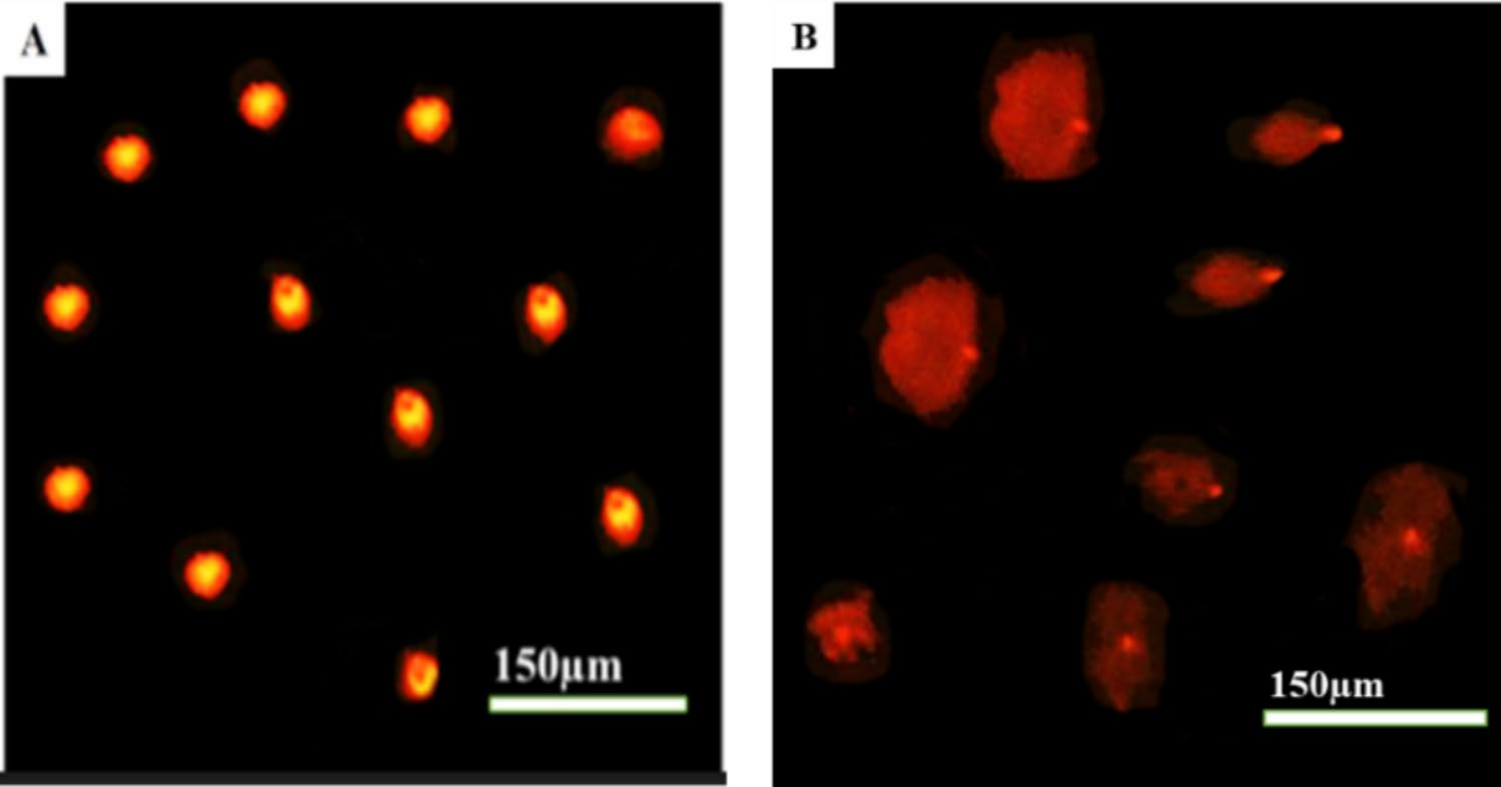
Comet assay photomicrographs showing the extent of DNA damage in liver tissue of pups of control and LNZ-treated dams. **A** Control group showing intact DNA. **B** Low dose group showing fragmented DNA

The liver tissue of low dose LNZ maternally treated pups showed a significant (*P* < 0.05) degree of DNA damage presented by a significant (*P* < 0.05) increase in the percentage of DNA damage, tail length, DNA percentage in the tail, and tail moment when compared to control group (Fig. 3, Table 7).

**Table 7:** The comet assay parameters of control and linezolid (LNZ) groups

**Parameter/group**	**Control**	**LNZ** (15.41mg/kg**)**
DNA damage %	9.1 ± 0.33	16.85 ± 1.25^a^
Tail length	7.47 ± 0.42	12.15 ± 0.74^a^
DNA % in tail	7.68 ± 0.32	9.61 ± 0.35^a^
Tail moment	0.63 ± 0.03	1.10 ± 0.12^a^

Each value represented as means ± SEM

^a^Letter means there was a significant difference (*P* < 0.05) as compared with control group. *n* = 36

## Discussion

Nursing mothers frequently receive medication for bacterial infections likely to be transferred to their infants during lactation (Begg et al. 2002; Tamburini et al. 2016). The exposure of newborns to drugs is of great concern due to the inadequate development of their liver and kidneys, which are responsible for processing and eliminating drugs, hence predisposing them to drug accumulation and adverse consequences (Begg et al. 2002; de Sá Del Fiol et al. 2016). Based on recent reports suggesting that risk of linezolid use varies with the age and condition of the examined population (Li et al. 2024; Abouelkheir et al. 2024), this study aimed to investigate the potential detrimental effects of the maternal LNZ administration during breastfeeding on Wistar rat’s offspring.

In the present investigation, the therapeutic dose of LNZ administered to lactating rats resulted in death of both mothers and their pups after four days of oral administration, whereas no mortality was observed during LNZ treatment at a dose of 15.41 mg/kg among lactating dams or their pups. This aligns with prior research, which reported that LNZ administration to female rats during gestation and lactation increased the risk of post-implantation embryonic mortality and pups’ death during early postnatal days (Hillard 2002; Vardakas et al. 2009).

Compared to the control group, we recorded a substantial weight loss in the low dose LNZ-treated mothers. Bobylev et al. (2016) revealed that LNZ-treated mice showed a slight reduction in body weight after 4 weeks. The weight loss in LNZ-treated dams concurs with the findings of Wang et al. (2014), who demonstrated that the oral administration of LNZ to rats at doses of 100 and 250 mg/kg/day for 15 days resulted in reduced food and water intake in the treated groups compared to the control groups. In the current study, LNZ administration also caused a significant decrease in pups’ body weight during the lactation period. Similarly, maternal mice treated with LNZ during gestation showed a reduction in the body weight of their fetuses (Vardakas et al. 2009). In line with our study, Tulstrup et al. (2018) revealed that exposure of lactating dams to either amoxicillin or vancomycin resulted in persistent loss in body weight of their pups. A clinical study conducted by Uzan-Yulzari et al. (2021) demonstrated that neonatal antibacterial drug exposure impairs normal growth in children during the first six years of life by perturbing intestinal microbial colonization.

Excessive ROS generation depletes the endogenous antioxidants, which subsequently fail to counteract ROS, resulting in cellular injury (Jadeja et al. 2017). The liver is a particularly vulnerable organ to ROS produced during xenobiotic biotransformation. Redox imbalance affects liver function, alters inflammatory pathways, and results in diseases (Allameh et al. 2023). Lipid peroxidation, initiated by free radicals, compromises the integrity of hepatocyte cell membrane and other peripheral tissues and causes a broad spectrum of damage (Skottová and Krecman 1998). SOD is the first enzyme in the antioxidant defense mechanism. It scavenges the superoxide radical and converts it into hydrogen peroxide. Catalase protects the cell from oxidative damage induced by hydrogen peroxide and hydroxyl radicals (Nandi et al. 2019; Rehman et al. 2020). GSH is a key factor in maintaining the cellular redox balance by protecting cells against exogenous and endogenous toxins, including ROS (Georgiou-Siafis and Tsiftsoglou 2023).

Our results showed a significant increase in MDA accompanied by a significant decrease in SOD, CAT, and GSH in the hepatic tissue of pups from the low dose LNZ-treated mothers compared with those of control mothers. Similar results were obtained by Wang et al. (2014), Vivekanandan et al. (2018), and Abd El Latif et al. (2019), who investigated the effect of LNZ administration on oxidative stress and antioxidant capacity in rats. Their results showed a significant increase in MDA with a significant decrease in antioxidant enzymes in the blood and liver of LNZ-treated rats, indicating that LNZ exposure increased lipid peroxidation and depletes the antioxidant reserve, hence triggering oxidative stress. LNZ-treated human patients also showed higher plasma MDA levels along with low levels of SOD and CAT (Wang et al. 2016). However, Kendir-Demirkol et al. (2023) demonstrated the elevation of MDA levels and antioxidant enzyme activities (SOD, CAT, GPx) in the blood of pediatric male rats after LNZ administration for two weeks, suggesting that the antioxidant system was activated to remove free radicals from LNZ group.

Microscopic examination of the liver tissue from suckling pups in the LNZ-treated dams revealed loss of hepatocyte architecture with marked damaged hepatocytes area, moderate congestion and infiltration. In an earlier study, infected rats treated with LNZ (50 mg/kg/twice/day) for 14 days revealed third-grade macrovesicular steatosis, two-thirds of the hepatocytes populations contained microvesicles of fat, apoptotic hepatocytes with diffuse vacuolization of hepatocytes coupled with grade two inflammation (Vivekanandan et al. 2018). Congestion of the hepatic portal blood vessels with moderate round cells infiltration and biliary proliferation were also detected in liver tissue of LNZ-treated rats (Abd El Latif et al. 2019). De Bus et al. (2010) revealed that prolonged linezolid treatment may result in significant hepatotoxicity. In a case study, the liver biopsy of a 55-year-old Caucasian woman revealed microvesicular steatosis with simultaneous acute liver failure and lactic acidosis following a 50-day LNZ treatment for an infected hip prosthesis. LNZ toxicity was considered to be the cause of lactic acidosis (De Bus et al. 2010), which caused damage to organs such as the liver and kidneys (Vivekanandan et al. 2018).

The histopathological examination of the renal tissue in the LNZ-treated group revealed degenerated, atrophied glomeruli and damaged renal tubules in the pups. Hyperplasia and loss of tubular epithelial Lining were also detected. In line with our work, rats treated with 100 mg/kg LNZ for 14 consecutive days suffered from mild interstitial nephritis, congestion of blood vessels, aggregates of inflammatory cells, and expansion of the tubular epithelium (Abdel Aziz et al. 2017; Hindawy and Hendawy 2019). Abd El Latif et al. (2019) recorded similar histopathological findings along with perivascular edema, varying degrees of hemorrhages, dilated collecting tubules and degenerated renal tubular epithelium with necrotic changes. Oxidative-induced renal damage has been linked to increased lipid peroxidation and low levels of antioxidant reserve, ultimately leading to cellular death; this may be responsible for the significant histopathological changes observed in the kidney of pups recorded in our study (Amini et al. 2022).

DNA is a highly sensitive genetic material to oxidative stress. DNA fragmentation may result from free radical attack during oxidative imbalance (Amin and Hamza 2005; Halliwell and Gutteridge 2015). In the present study, comet assay revealed a significant DNA fragmentation in pups’ liver tissue maternally treated with 15.41 mg/kg LNZ during lactation period. Our results suggest that the oxidative stress induced by LNZ treatment may play an important role in DNA damage. This is congruent with the study by Amin and Hamza (2005), who found that drug-induced hepatotoxicity was accompanied by hepatic DNA fragmentation and lipid peroxidation (MDA). DNA fragmentation and lipid peroxidation seen in their study are consequences of oxidative stress, as evidenced by reduced GSH and SOD activities in the liver.

## Conclusion

The present study demonstrated that administering the therapeutic dose of LNZ to lactating mothers has lethal effects. This study also revealed that mothers who received a low dose of LNZ during lactation induced redox imbalance in their offspring, resulting in oxidative stress and DNA damage. In addition, the hepatic and renal tissues of the pups showed histopathological changes after maternal LNZ treatment. The present work reveals the health consequences of exposure of rat pups to LNZ through lactation and recommends that physicians should use caution when administering this antibacterial drug to nursing women, and only when the benefit clearly justifies the potential risk to the offspring.

### Limitations and future plans

This work provides information on the adverse effects of LNZ on the health of pups during the lactation period. Future research is needed to assess the safety of various sub-therapeutic dosages and durations of exposure to provide appropriate guidelines for linezolid use during lactation. Furthermore, further studies are required to investigate its impacts during post-weaning and adult stages. Filling these research gaps will help us better understand the health issues related to LNZ exposure and develop appropriate risk-reduction strategies.

## Data Availability

All source data for this work (or generated in this study) are available upon reasonable request.
